# Polymerization of a resin composite luting agent: effects of temperature, preheating duration, and light-curing duration

**DOI:** 10.3389/froh.2026.1881185

**Published:** 2026-07-09

**Authors:** Ahmad Alsahli, Syed Saad B. Qasim, Dalal AlMatar, Shaikha AlSanousi, Mirza Rustum Baig

**Affiliations:** 1 Department of General Dental Practice, College of Dentistry, Kuwait University, Safat, Kuwait; 2 Department of Bioclinical Sciences, College of Dentistry, Kuwait University, Safat, Kuwait; 3 Ministry of Health, Safat, Kuwait; 4 Department of Restorative Sciences, College of Dentistry, Kuwait University, Safat, Kuwait

**Keywords:** composite resins, degree of conversion, light cure, microhardness, polymerization, preheated composite, resin cements

## Abstract

**Objective:**

This study evaluated how preheating temperature and duration, and light-curing time affect the microhardness (HV) and degree of conversion (DC) of a preheated resin composite used as a luting agent.

**Materials and methods:**

A microhybrid composite was preheated to 54 °C or 68 °C for 5, 10, or 15 min, and then placed between a 4.0 mm lithium disilicate overlay and a natural tooth as a luting agent. Light-curing was performed for 120, 180, or 270 s. The microhardness and degree of conversion were measured. Three-way ANOVA analyzed the effects and interactions of the variables. Pearson's correlation assessed the relationship between HV and DC.

**Results:**

DC was not significantly affected by preheating temperature, duration, or light-curing time (*p* > 0.05). HV was significantly influenced by all variables (*p* ≤ 0.001), with the highest HV at 68 °C for 15 min and longer curing. A significant three-way interaction was noted (*p* = 0.039). No consistent correlation was found between DC and HV, except for one condition (54 °C, 5 min, 90 s; *ρ* = 0.97, *p* = 0.005).

**Conclusions:**

Within the preheating conditions evaluated, higher preheating temperatures, longer preheating durations, and extended curing times were associated with increased microhardness, but not the degree of conversion, of the resin composite under ceramic restorations.

**Clinical significance:**

Among the preheating protocols evaluated, higher preheating temperatures, longer preheating durations, and extended multidirectional light-curing times were associated with higher microhardness of resin composites used as luting agents beneath thick (4 mm) lithium disilicate restorations, without affecting the degree of conversion. Because no non-preheated control group was included, these findings should be interpreted as comparisons among different preheating protocols rather than evidence of the benefits of preheating itself. Furthermore, the extended curing times investigated in this study represent optimal experimental conditions for the evaluated restoration configuration and may not always be practical or feasible in routine clinical settings. Minimizing the delay between preheating and curing may help preserve the effects associated with the investigated preheating protocols.

## 1 Introduction

Advances in dental materials and adhesive techniques have shifted the direction of dentistry toward minimally invasive, lesion-based treatment approaches that prioritize the preservation of healthy tooth structure by removing only diseased tissues. Ceramic indirect restorations, including inlays and onlays, exemplify this approach by restoring localized defects and minimizing the need for extensive tooth reduction associated with full-coverage crowns (1, 2). The clinical success of these restorations is influenced in part by the choice of luting agent. Restorative resin composites (RCs) have been proposed as an alternative to conventional resin cement due to their superior mechanical properties, fracture strength, and dentinal bond strength (3–6). However, their high viscosity can hinder the seating of indirect restorations, thus making them an undesirable option as a luting agent (7). The challenge of achieving optimal polymerization, essential for better mechanical properties and long-term retention, remains a concern, particularly for thick indirect restorations (8). Consequently, clinicians usually default to using dual-cured resin cements, despite emerging evidence casting doubt on this preference (9–11).

The polymerization of RCs in ceramic restorations, as measured by degree of conversion (DC) and microhardness (HV), is influenced by factors such as ceramic thickness, shade, translucency, and opacity (12–14). Studies have shown that RCs may be adequately polymerized under indirect restorations with thicknesses of 7.5–9.5 mm, provided sufficient light-curing irradiation is used (12, 15).

The power and exposure time of light-curing irradiation—determinants of the total irradiation energy —have a direct impact on RC polymerization (16). While different combinations of irradiation power and exposure time can be used to achieve the manufacturer-recommended irradiance, using higher irradiance for shorter durations may increase the shrinkage stress rate of resin composites (17, 18). Conversely, prolonging the exposure time could potentially damage the pulp and surrounding soft tissues (19). The goal is to achieve adequate polymerization, especially when placing multiple restorations, while ensuring the health of the pulp and soft tissues (12, 20).

Preheating RCs prior to direct light-curing activation has been shown to decrease the required exposure time, improve DC, and decrease viscosity (21, 22). An elevation in temperature has been demonstrated to enhance the movement of molecules, thereby reducing viscosity and increasing the rate of species collision. This phenomenon facilitates increased monomer conversion before autodeceleration; hence, more monomers will then polymerize before vitrification. As monomer conversion increases, the glass transition temperature of the reacting mixture will rise, allowing higher polymerization temperatures and a higher final limiting conversion (23, 24). These thermal and kinetic effects may optimize polymer network formation, leading to enhanced mechanical properties and curing efficiency.

The optimal preheating temperature (PT) and preheating duration (PD) are still debated. Proposed PTs range from 30 °C to 69 °C, and it was shown that certain PTs influenced microleakage, marginal adaptation, and cellular toxicity (25–28). Similarly, PDs have varied widely in the literature, from 40 s to 24 h. Concerns have been raised regarding potential cellular toxicity from excessive preheating beyond certain temperatures (28).

Although RCs used as luting agents are often preheated to reduce viscosity and facilitate restoration seating, differences between PTs of 54 °C and 68 °C under thick indirect restorations have yet to be investigated, and a clear understanding of the role of PT and PD is lacking (17, 29, 30). Previous studies have tested polymerization RCs as luting agents under simulated conditions, but did not preheat the RCs or replicate clinical situations, such as anatomical crown restoration or multidirectional curing (10, 12, 15, 31, 32). In the present study, a 4.0 mm thick ceramic restoration was selected because it represents the occlusal thickness commonly encountered in extensive indirect posterior restorations, where light transmission may be substantially attenuated. Although the preheating of RCs has shown laboratory benefits, it remains unclear how different preheating temperatures, durations, and light-curing times affect polymerization under thick restorations, simulating real clinical conditions.

Therefore, this *in vitro* study aims to assess preheated RC polymerization under a 4.0 mm thick ceramic restoration placed on a natural tooth preparation. The effects of different preheating temperatures and durations and light-curing times on DC and HV were assessed. The following null hypotheses were established for this study: (1) preheating temperature and duration or light-curing time would not affect the DC of RCs; (2) preheating temperature and duration or light-curing time would not affect the HV of RCs; and (3) no association would be observed between the DC and HV of RCs across all investigated variables.

## 2 Materials and methods

This study investigated the effects of preheating temperature (PT), preheating duration (PD), and light-curing (LC) duration on the degree of conversion (DC) and microhardness (HV) of restorative resin composites.

### 2.1 Ethical approval and sample source

**Figure 1:**
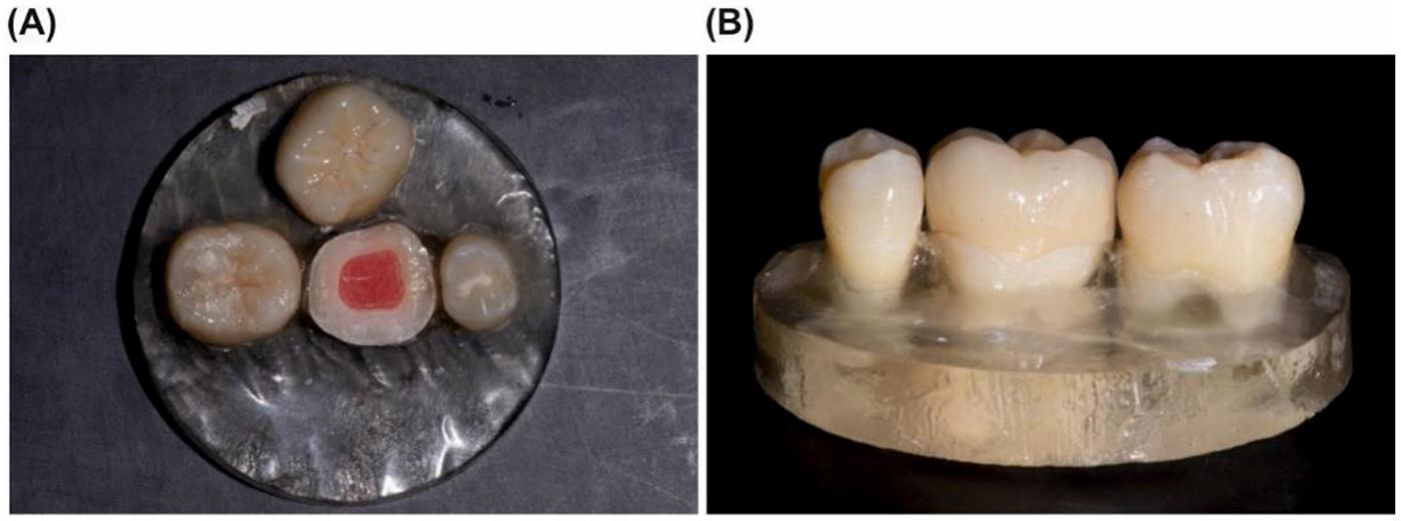
**(A)** occlusal and **(B)** lingual views of the dental mold and overlay used in the study.

**Figure 2:**
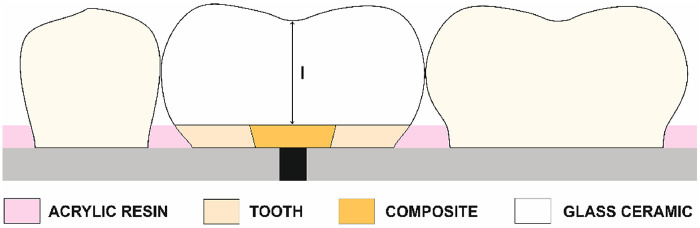
Experimental mold showing the overlay thickness (I: 4.0 mm) from the central grooves to the composite luting agent chamber, which has an injection channel at the bottom (adopted and modified from de Kuijper et al. (10).

Ethical approval was obtained (Approval No. VDR/EC-464). No participant consent was required as the study used an extracted natural tooth. A sound, caries-free single mandibular first molar, extracted for therapeutic reasons, was embedded with an adjacent second molar and second premolar in an acrylic resin block (Figures 1, 2).

### 2.2 Tooth preparation and ceramic overlay design

The embedded arch was scanned using a Trios 3 intraoral scanner (3Shape, Copenhagen, Denmark). An overlay preparation was performed on the mandibular first molar with a minimum thickness of 4.0 mm at the central groove, verified using a putty matrix (Figure 1). A luting agent box was prepared at the center of the occlusal surface (Figures 1, 2) (10). The box measured 5.0 mm mesiodistally and 4.0 mm buccolingually. An access opening to the pulp chamber was created at its base, serving as an ejection tunnel for the retrieval of the resin composite (RC) (33). The inner walls were lubricated, and a self-curing resin plug (Pattern Resin, GC, United States) was placed to standardize the depth to 1.5 mm. The box was sealed with peripheral wax (Yeti Dental, Engen, Germany), and a second scan was recorded.

### 2.3 Ceramic fabrication

Two scans were used to design and fabricate a single pressed lithium disilicate ceramic overlay [12.0 mm mesiodistally × 10.75 mm buccolingually; A2 shade, low translucency (LT); Ivoclar Vivadent, Schaan, Liechtenstein] (Table 1). The same overlay was used with all samples in the study, for standardization purposes.

**Table 1:** Materials used in the study.

Batch number	Manufacturer	Composition	Filler loading	Type	Brand
T29853	Ivoclar Vivadent, Schaan, Liechtenstein	SiO_2_, Li_2_O, K_2_O, MgO, ZnO, Al_2_O_3_, P_2_O5 and other oxides^a^		Lithium Disilicate glass ceramic	IPS e.max Press, LT A2
980791, 2N0781, 5C0785	Kuraray Noritake Dental	Bis-GMA, TEGDMA, silanated barium glass filler, silanated silica filler, silanated colloidal silica, DL-camphorquinone	86 wt%/70 vol%	Microhybrid light-cured restorative composite, shade A2	Clearfil™ AP-X PLT

a Manufacturer information.

### 2.4 Sample size determination

The sample size was determined using G*Power software (version 3.1) for a three-way fixed-effects ANOVA design (PT  ×  PD  ×  LC; 18 groups total). The effect size was estimated from Vickers hardness values reported by D'Arcangelo et al. (31) for resin composite luting agents polymerized through 4.0 mm ceramic overlays across three curing times. Cohen's f was calculated from the standard deviation of group means and the pooled within-group standard deviation, yielding *f* = 2.35 (partial *η*^2^ = 0.847). With an alpha level of.05 and a target power of 95%, the minimum estimated sample size was two specimens per group. This was rounded up to five specimens per group in accordance with similar studies in the field (31, 34), yielding a total sample of 90 specimens.

### 2.5 Composite preheating and placement

**Figure 3:**
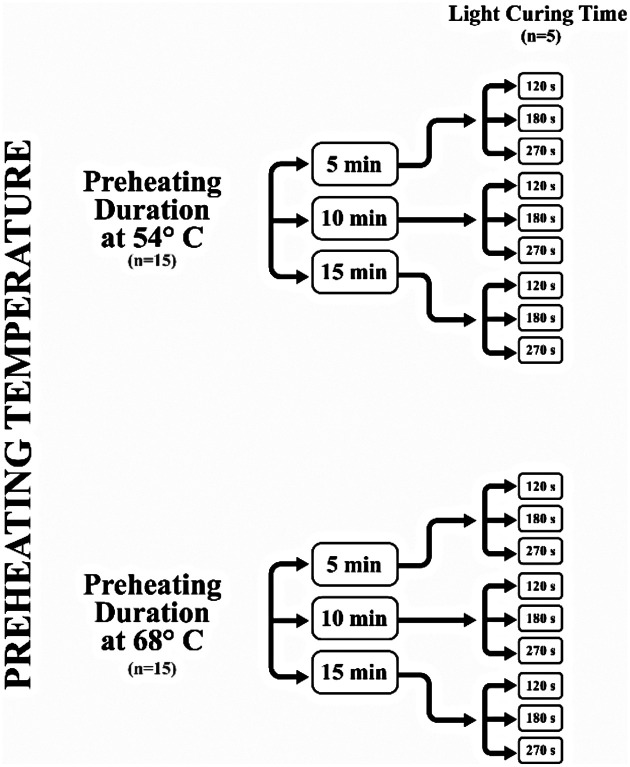
Flowchart of the experimental conditions.

The experimental design comprised six groups of microhybrid composites (shade A2, AP-X; Kuraray Noritake Dental, Okayama, Japan) in preloaded tips (*n* = 15 per group) (Table 1). A microhybrid resin composite was selected because this category of materials is well characterized, possesses high filler loading, and has extensively documented physical and mechanical properties. The use of a microhybrid composite enabled reliable evaluation of the effects of the preheating and light-curing protocols on resin composite polymerization while facilitating comparison with previous studies investigating similar variables. The composite was preheated in a Calset heating unit (AdDent Inc., Danbury, CT, United States) at either 54 °C (T54) or 68 °C (T68) for 5, 10, or 15 min (Figure 3). Temperature stability was verified by placing a thermometer inside the unit. After preheating, the composite was dispensed into the luting agent box on top of the removable Pattern Resin stop, which had previously been covered with Teflon tape. Excess composite was removed, a Mylar strip was placed, and the ceramic overlay was seated on the preparation (Figure 1).

### 2.6 Light-Curing protocol

Each sample was light-cured for 40, 60, or 90 s from the lingual, occlusal, and buccal directions (*n* = 5), resulting in total curing times of 120, 180, or 270 s per sample (Figure 3). A light irradiance of 1,000 mW/cm² was applied using a VALO Grand LED curing unit (Ultradent Products Inc., South Jordan, UT, United States) operating in Standard Power mode (wavelength range: 385–515 nm). The irradiance was verified with a Cure Rite radiometer (Dentsply Caulk, Milford, DE, United States) to ensure a minimum output of 850 mW/cm^2^ for each subgroup. During light curing, the curing tip was positioned approximately 1–2 mm from the ceramic surface to avoid displacement of the seated restoration. The interval between the removal of the composite from the preheating unit and the initiation of light curing was standardized and did not exceed 60 s.

### 2.7 Sample removal and grouping

After light curing, the ceramic onlay was removed with gentle pressure, and the resin composite (RC) disk was ejected from the box by inserting a thin rod through the pulp chamber. Two RC disks were prepared for each experimental condition: one for degree of conversion (DC, S1) and one for microhardness (HV, S2). The disks were approximately 1.5 mm thick. The light-exposed surface of each disk was marked immediately to ensure consistent orientation during HV testing.

To minimize potential confounding variables, all groups were prepared and tested under standardized conditions, including identical resin composite material and shade, ceramic material and thickness, specimen dimensions, preheating procedures, curing unit, and testing protocols.

### 2.8 DC analysis

Once the samples had been pulverized (*n* = 5), the degree of conversion (DC) was evaluated using Fourier transform infrared spectroscopy (FTIR; Tensor 27, Bruker Optics GmbH, Ettlingen, Germany) in attenuated total reflectance (ATR) mode. The entire specimen was fully pulverized before FTIR-ATR analysis; therefore, the calculated DC represents the average bulk degree of conversion for each specimen. The specimens were placed on a diamond crystal, and spectra were acquired with OPUS software (version 6.5) in the mid-infrared region (400–4,000 cm^−1^) at a resolution of 4 cm^−1^ with 32 scans per specimen. A background spectrum was collected prior to analysis to eliminate atmospheric water vapor and CO_2_, and was repeated every 30 min to ensure accurate subtraction. The spectra were processed using OMNIC 9 software (Thermo Fisher Scientific, Waltham, MA, United States).

The ratio of absorbance peak heights at 1,638 cm−1 (aliphatic C = C) and 1,608 cm^−1^ (aromatic C = C) was measured for uncured and cured specimens. The percentage of unreacted double bonds (%DC) was calculated using the following formula:

$$\mathrm{DC}\;(\text{\%})=1-\frac{\left((1638\;cm^{-1}/1608\;cm^{-1})\;\mathrm{cured}\right)}{\left({\left(1638\;cm^{-1}/1608\;cm^{-1}\right)}^{\;}\mathrm{uncured}\right)}\;\times \;100\text{\%}$$

### 2.9 Microhardness

The light-cured side of each S2 disk was marked, embedded in epoxy resin, and polished with silicon carbide paper (1,000-grit, CarbiMet, Buehler Ltd., Lake Bluff, IL, United States) for 90 s under intermittent manual pressure. After storage for 7 days (35), the specimens were positioned perpendicular to the indenter tip, and Vickers microhardness (HV) was measured using a digital tester (CV-400DAT, CV Instruments Ltd., United Kingdom) equipped with a Vickers diamond indenter. A load of 100 g was applied for 10 s. Five random indentations were made per specimen, and the diagonal lengths were measured with a 10 ×  objective lens.

Microhardness (HV) was calculated using the following formula:

HV=1,854.4P/d2

where P is the applied load (g) and d is the mean diagonal length of the indentation (µm).

### 2.10 Statistical analysis

To investigate the effects of PT, PD, and LC duration on the DC and HV of restorative RCs, the experimental design followed a fully crossed 2 × 3 × 3 between-subject factorial structure consisting of two temperature levels (54 °C and 68 °C), three preheating durations (5, 10, and 15 min), and three light-curing durations: 120 s (3 × 40), 180 s (3 × 60), and 270 s (3 × 90). Each experimental condition comprised five independent specimens (*n* = 5), yielding 90 observations for DC and 90 for HV (18 groups × 5 specimens). One measurement was recorded per specimen for DC, while five HV readings were averaged into a single value per specimen.

Separate three-way factorial analyses of variance (ANOVAs) were conducted for DC and HV. Prior to analysis, assumptions of homogeneity of variances and normality were evaluated. Levene's test confirmed equal variances across groups for both outcomes. The normality of residuals was assessed within each cell using Shapiro–Wilk tests and Q-Q plots, which indicated no substantial departures from normality. Given the balanced sample sizes, the ANOVA procedure was considered robust to minor deviations. All main effects, two-way interactions, and the three-way interaction were included in the model. When statistically significant effects were identified, Bonferroni-adjusted pairwise comparisons of estimated marginal means were performed to explore group differences.

Spearman's rho correlation analyses were conducted to explore the association between HV and DC across experimental conditions. All statistical analyses were conducted using IBM SPSS Statistics for Windows, Version 29.0 (IBM Corp., Armonk, NY, United States), with the significance level set at *α* = .05.

## 3 Results

### 3.1 Degree of conversion (DC)

A three-way ANOVA was conducted to evaluate the effects of PT, PD, and LC duration on DC. None of the main effects were significant: PT, F(1, 72) = 0.001, *p* = 0.973; PD, F(2, 72) = 1.55, *p* = 0.220; and LC, F(2, 72) = 1.27, *p* = 0.288. Similarly, no significant two-way interactions were found: PT × PD, F(2, 72) = 2.21, *p* = 0.117; PT × LC, F(2, 72) = 1.74, *p* = .183; and PD × LC, F(4, 72) = 1.16, *p* = 0.335. The three-way interaction was also nonsignificant: F(4, 72) = 0.60, *p* = 0.666. (Table 2).

**Table 2:** Effect of temperature, preheating duration, and light-curing duration on the degree of conversion (DC) of restorative resin composites.

Temperature	LC120		LC180		LC270	
	*M*	*SD*	*M*	*SD*	*M*	*SD*
Group: T54						
5 min	86.54	5.69	90.54	4.11	90.53	1.86
10 min	85.82	2.65	87.81	8.98	89.02	4.52
15 min	83.60	7.44	83.04	4.71	88.02	1.42
Group: T68						
5 min	84.32	3.44	86.18	2.06	88.97	7.86
10 min	90.36	1.78	87.59	3.20	86.38	3.11
15 min	88.98	3.02	85.35	2.72	87.07	5.76

Note. *M*, mean; *SD*, standard deviation; LC, light-curing duration in seconds. The table presents the mean and standard deviation of the degree of conversion (DC) for restorative resin composites preheated at 54 °C (T54) and 68 °C (T68) for varying durations (5, 10, and 15 min) under different light-curing durations (120, 180, and 270 s). Each mean value is based on five independent specimens tested under a unique combination of preheating temperature, preheating duration, and light-curing time.

**Figure 4:**
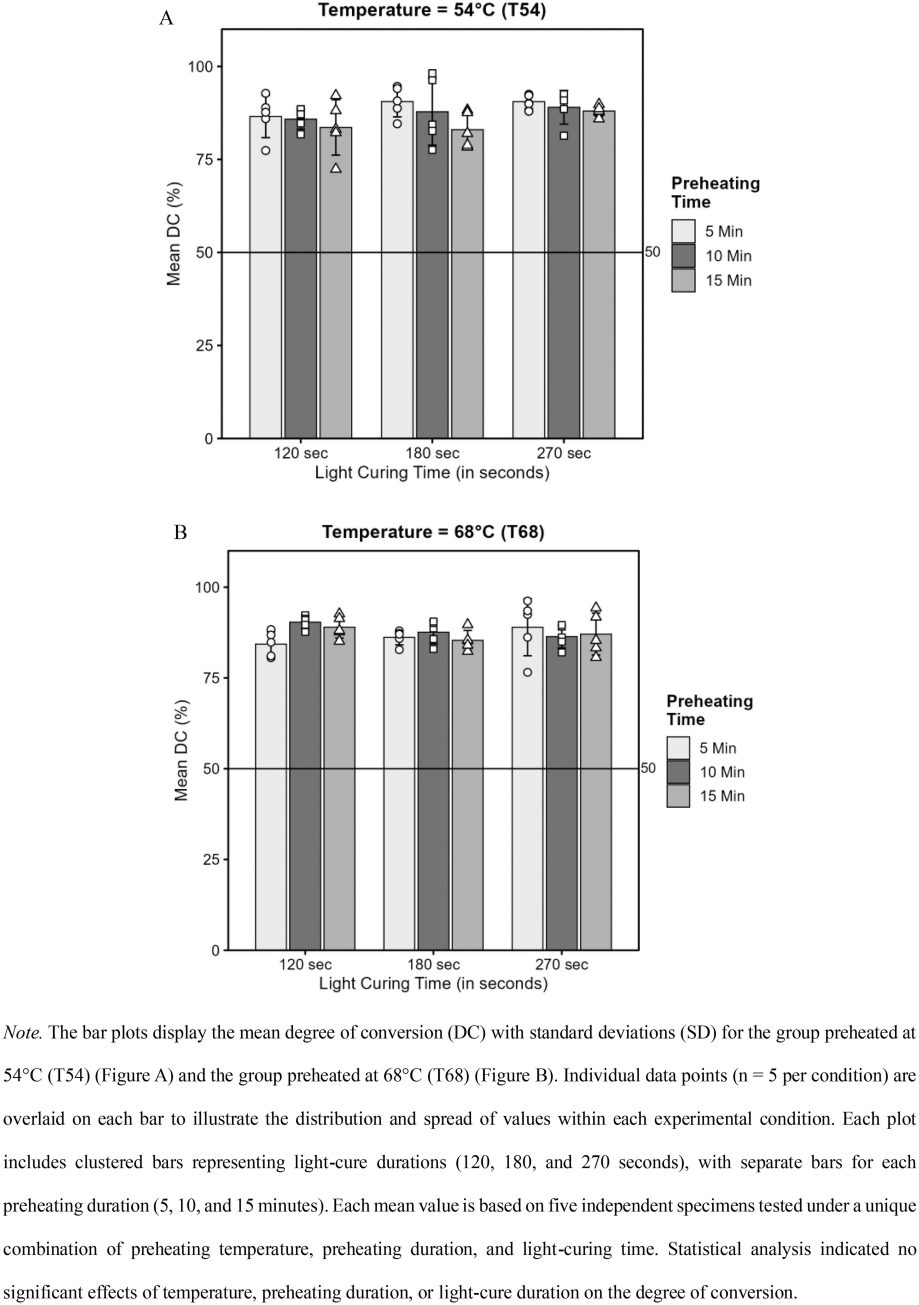
Bar plots of degree of conversion (DC) of restorative resin composites: effects of temperature, preheating duration, and light-curing duration.

The bar plots in Figure 4 illustrate the degree of conversion (DC) of resin composites across different preheating durations and light-curing times at 54 °C (A) and 68 °C (B). In both temperature groups, DC values remained consistent across all experimental conditions, with means clustered closely and no observable trends. These visual findings align with the statistical results, confirming that PT, PD, and LC within the tested ranges did not have a significant effect on DC.

### 3.2 Vickers microhardness (HV)

A three-way factorial ANOVA revealed significant main effects of PT, PD, and LC time on HV (Table 3). The main effect of temperature was significant; *F*(1, 72) = 57.23, *p* < 0.001, partial *η*^2^ = 0.443. Estimated marginal means showed that specimens preheated at 68 °C exhibited significantly higher HV values (M = 122.02, SE = 1.04) than those preheated at 54 °C (M = 110.89, SE = 1.04), with a Bonferroni-adjusted mean difference of 11.12, *p* < 0.001. The main effect of preheating duration was also significant; *F*(2, 72) = 38.27, *p* < 0.001, partial *η*^2^ = 0.515. Bonferroni-adjusted comparisons indicated that specimens preheated for 15 min (M = 125.53, SE = 1.27) had significantly higher HV than those preheated for 5 min (M = 111.37, SE = 1.27) and 10 min (M = 112.47, SE = 1.27); both *p* < 0.001. No significant difference was observed between 5 and 10 min: *p* = 1.000. The main effect of light-curing duration was also statistically significant; *F*(2, 72) = 7.55, *p* = 0.001, partial *η*^2^ = 0.173. Bonferroni-adjusted comparisons showed that HV values at 40 s (M = 112.48, SE = 1.27) were significantly lower than at 60 s (M = 119.07, SE = 1.27), *p* = 0.001, and 90 s (M = 117.81, SE = 1.27), *p* = 0.012, while no significant difference was observed between 60 and 90 s; *p* = 1.000.

**Table 3:** Effect of temperature, preheating duration, and light-curing duration on the microhardness (HV) of restorative resin composites.

Temperature	LC120	LC180	LC270
	M ± SD	M ± SD	M ± SD
Group: T54			
5 min	100.70 ± 12.18^a^	117.76 ± 4.56	110.88 ± 7.63
10 min	111.02 ± 8.46	114.44 ± 7.31	110.44 ± 3.84
15 min	106.41 ± 5.27^a^	114.80 ± 3.26^a^	111.60 ± 2.38^a^
Group: T68			
5 min	114.28 ± 2.39^b^	113.72 ± 8.81	110.86 ± 8.97
10 min	112.44 ± 5.66	112.32 ± 5.94	114.16 ± 3.75
15 min	130.04 ± 7.60^b^	141.40 ± 11.97^b^	148.92 ± 4.23^b^

Note. M, mean; SD, standard deviation; LC, light-curing duration in seconds. The table presents the mean and standard deviation of microhardness (HV) for restorative resin composites preheated at 54 °C and 68 °C for varying durations (5, 10, and 15 min) under different light-curing durations (120, 180, and 270 s). Each mean value is based on five independent specimens tested under a unique combination of preheating temperature, preheating duration, and light-curing time. Within each preheating duration × curing condition, values with different superscripts (^a^, ^b^) differ significantly at *p* < 0.05 (Bonferroni-adjusted). Cells without superscripts did not differ significantly between temperature groups.

Regarding the two-way interactions, only the interaction between PT and PD was statistically significant; *F*(2, 72) = 37.92, *p* < 0.001, partial *η*² = 0.513. Simple effects analysis revealed that this interaction was primarily driven by the 15-minute condition, where the 68 °C group had significantly higher HV values (M = 140.12, SE = 1.80) than the 54 °C group (M = 110.94, SE = 1.80), with a mean difference of 29.18 *(p* < 0.001). No significant differences in temperature were observed at 5 min (*p* = 0.217) or 10 min (*p* = 0.694). The two-way interaction between temperature and light-curing duration was not statistically significant, *F*(2, 72) = 2.17, *p* = 0.121, partial *η*^2^ = 0.057, nor was the interaction between preheating duration and light-curing duration; *F*(4, 72) = 2.30, *p* = 0.067, partial *η*^2^ = 0.113.

The three-way interaction between temperature, preheating duration, and light-curing duration was statistically significant; *F*(4, 72) = 2.66, *p* = 0.039, partial *η*^2^ = 0.129. Bonferroni-adjusted comparisons revealed that, at the 15-minute condition, the 68 °C group showed significantly higher HV values than the 54 °C group across all curing times (40 s: mean difference = 23.63, *p* < 0.001; 60 s: mean difference = 26.60, *p* < 0.001; 90 s: mean difference = 37.32, *p* < 0.001). A smaller but statistically significant difference was also observed at 5 min and 40 s (mean difference = 13.58, *p* = 0.003). No other pairwise comparisons within the 5- or 10-minute groups reached significance. In Table 3, these differences are denoted by different superscripts.

**Figure 5:**
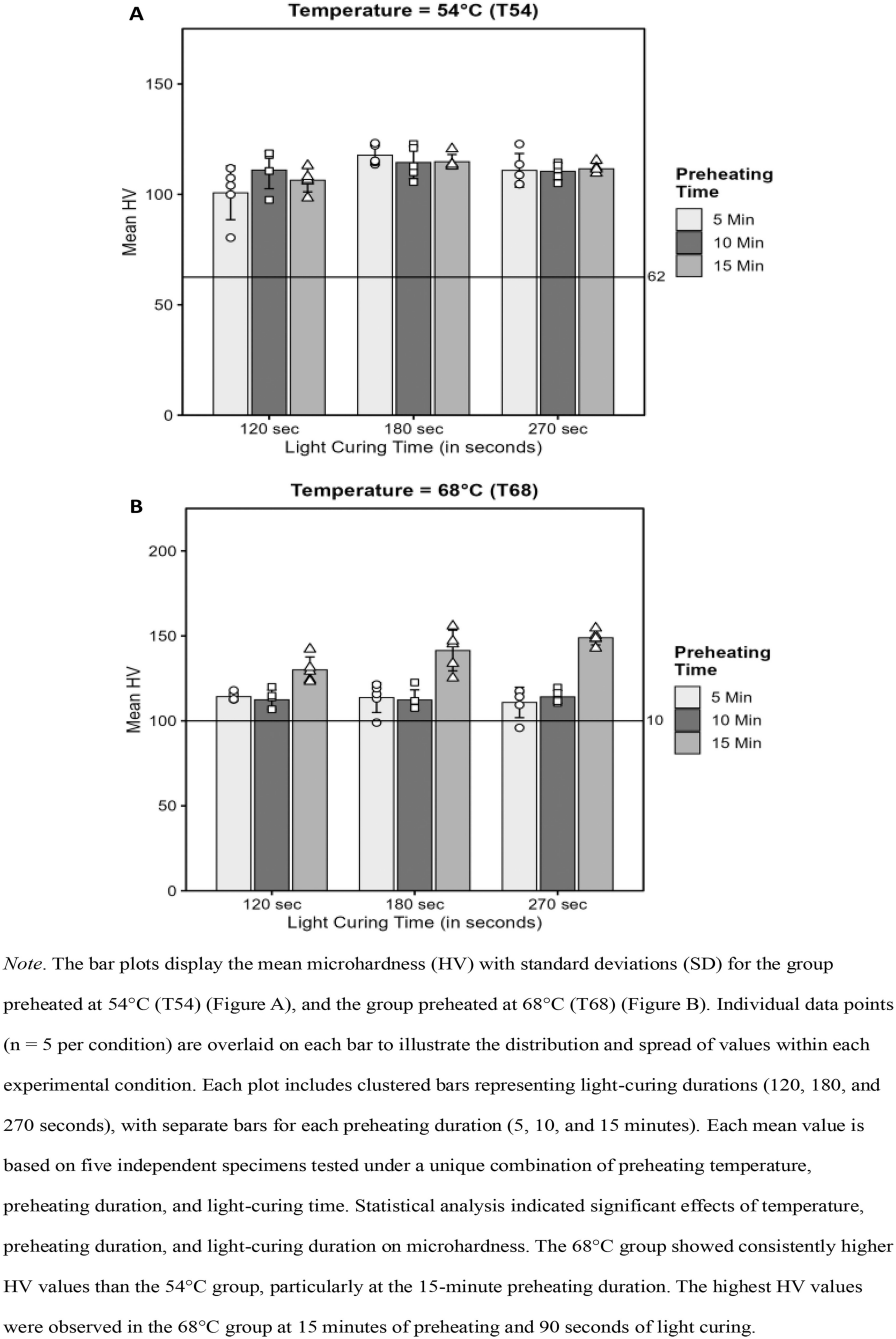
Bar plots of degree of microhardness (HV) of restorative resin composites: effects of temperature, preheating duration, and light-curing duration.

Figure 5 illustrates the microhardness (HV) of restorative resin composites under varying conditions of PT, PD, and LC duration. In the 54 °C group (Figure A), HV values remained relatively consistent across preheating and light-curing durations, showing only modest variation. In contrast, the 68 °C group (Figure B) displayed a clear trend of increasing HV with longer preheating times and extended light-curing exposure. The most pronounced increases in HV were observed at 15 min of preheating across all light-curing durations, consistent with the significant main effects and interaction results. Notably, at 15 min, the 68 °C group achieved substantially higher HV values than the 54 °C group across all curing durations (120, 180, and 270 s), with the largest differences observed at 270 s. These patterns visually support the statistical findings that temperature and preheating duration have a synergistic effect on microhardness within the range of preheating conditions evaluated, with the highest HV values observed under high-temperature, long-duration preheating conditions.

### 3.3 Correlation analysis

In the 54 °C group (T54), a significant positive correlation was found only in the condition with 5 min of preheating and 270 s of light curing [*ρ* = 0.975, *ρ^2^* = 0.95, 95% CI (0.64, 1.00), *p* = 0.005]. No significant correlations were observed under the other conditions (*ρ* range: −0.70 to 0.80, *p* > 0.05). In the 68 °C group (T68), no significant correlations were detected (*ρ* range: −0.80 to 0.41, *p* > 0.05). Confidence intervals for all subgroups are reported in Table 4.

**Table 4:** Correlation between microhardness (HV) and degree of conversion (DC) under different experimental conditions.

HV vs. DC				
Groups	*ρ*	*ρ*²	95% CI	*p*
T54				
LC: 120 s—D: 5 min	−0.30	0.09	[−0.94, 0.81]	0.624
LC: 180 s—D: 5 min	0.20	0.04	[−0.84, 0.93]	0.747
LC: 270 s—D: 5 min	0.98**	0.95	[0.64, 1.00]	0.005
LC: 120 s—D: 10 min	0.36	0.13	[−0.78, 0.95]	0.553
LC: 180 s—D: 10 min	0.80	0.64	[−0.32, 0.99]	0.104
LC: 270 s—D: 10 min	−0.70	0.49	[−0.98, 0.51]	0.188
LC: 120 s—D: 15 min	−0.60	0.36	[−0.97, 0.63]	0.285
LC: 180 s—D: 15 min	0.20	0.04	[−0.84, 0.93]	0.747
LC: 270 s—D: 15 min	−0.21	0.04	[−0.93, 0.84]	0.741
T68				
LC: 120 s—D: 5 min	0.21	0.04	[−0.84, 0.93]	0.741
LC: 180 s—D: 5 min	0.10	0.01	[−0.87, 0.91]	0.873
LC: 270 s—D: 5 min	−0.80	0.64	[−0.99, 0.32]	0.104
LC: 120 s—D: 10 min	0.41	0.17	[−0.76, 0.95]	0.493
LC: 180 s—D: 10 min	−0.15	0.02	[−0.92, 0.85]	0.805
LC: 270 s—D: 10 min	−0.30	0.09	[−0.94, 0.81]	0.624
LC: 120 s—D: 15 min	−0.80	0.64	[−0.99, 0.32]	0.104
LC: 180 s—D: 15 min	−0.30	0.09	[−0.94, 0.81]	0.624
LC: 270 s—D: 15 min	0.10	0.01	[−0.87, 0.91]	0.873

N, 5. ρ, Spearman's correlation coefficient; ρ^2^, coefficient of determination (proportion of shared variance); 95% CI, confidence interval based on Fisher's z-transformation (Fieller, Hartley & Pearson method); LC, light-curing duration in seconds; D, preheating duration in minutes. The wide confidence intervals across all subgroups reflect the limited statistical precision inherent to correlations based on n, 5 per cell and should be interpreted with caution.

** *p* < 0.01.

## 4 Discussion

This study investigated the influence of preheating temperature (PT), preheating duration (PD), and light-curing (LC) time on the degree of conversion (DC) and microhardness (HV) and their correlation in a resin composite used as a luting agent beneath a 4 mm lithium disilicate restoration. The results showed that DC was not significantly affected by PT, PD, or LC, and none of the interaction terms reached significance. Thus, the first null hypothesis was retained. In contrast, microhardness (HV) was significantly influenced by all three main factors, PT, PD, and LC, with the most pronounced improvements observed at 68 °C and 15 min of preheating under light-curing for 180 or 270 s. These findings led to the rejection of the second null hypothesis. As for the third null hypothesis, it was not rejected, as no correlation was found between HV and DC under the experimental conditions. These results indicate that variations in preheating temperature, preheating duration, and curing protocols influenced RC microhardness without altering the degree of conversion.

In this study, DC was not significantly influenced by preheating temperature, duration, or light-curing time, and no interactions among these factors were observed. Previous research on the effect of preheating on RC DC has been heterogeneous (36–38); some studies reported improved DC with preheating (17, 21, 39), while others found no effect (31, 36, 40, 41). As our design did not include a non-preheated control group, the present findings specifically demonstrate that varying preheating conditions and curing times within preheated composites did not improve DC. A likely explanation is the rapid drop in composite temperature after the removal of the sample from the heating device (42, 43). Another contributing factor could be that preheating can affect the properties of resin composites, and the extent of this effect may vary among materials depending on their composition and filler content (44). Overall, DC remained stable across all tested thermal and photopolymerization conditions, supporting the robustness of this parameter under the studied ranges.

In contrast to DC, HV was significantly influenced by all tested factors. Preheating temperature had a significant effect, with 68 °C yielding higher HV values than 54 °C. Preheating duration also showed a significant effect, as the 15 min group exhibited higher HV values than both the 5- and 10-min groups. Longer light-curing times (180 and 270 s) also resulted in significantly higher HV values than the 120-second duration. Collectively, these results indicate that the combination of preheating at 68 °C for 15 min and extended light curing produced the highest HV values observed in this study.

The higher HV values observed under certain preheating conditions may be attributed to temperature-related effects on the material's molecular structure. Preheating resin composites enhances molecular mobility, increasing the collision frequency of reactive species and prolonging propagation before vitrification. This delays diffusion-controlled propagation and reaction–diffusion termination, thereby reducing autodeceleration and enabling higher monomer conversion and greater final conversion limits. Consequently, preheating to temperatures above room temperature can promote the formation of a more highly crosslinked polymer network, which may result in improved mechanical and physical properties (21, 45, 46).

However, the beneficial effect of preheating may dissipate once the composite is removed from the heating device (42). It can therefore be speculated that longer preheating durations and higher preheating temperatures could help offset this “cooling effect”, enabling more effective crosslinking before vitrification occurs.

This study was designed to simulate a clinical setting in which polymerization begins no later than 60 s after the RC is removed from the preheating device, based on studies showing a 50% drop in preheated RC temperature after 2 min of HV (42).

Some studies have shown that preheating RCs increases their HV (31, 36, 47), whereas others have found no improvement (37). This study shows that temperature and preheating duration have a synergistic effect on microhardness, with the highest HV values achieved under high-temperature, long-duration preheating conditions.

However, the effectiveness of polymerization is also constrained by light transmission through the ceramic restoration. Light attenuation through lithium disilicate significantly reduces the amount of irradiance reaching luting agents, necessitating extended curing protocols (31). Our results demonstrated that extended curing times (180–270 s) significantly improved HV compared to shorter exposures (120 s) when preheated RCs were used beneath 4 mm lithium disilicate restorations. Previous studies have confirmed that conventional curing times (20–120 s) are insufficient for thick ceramics (11, 12, 31), and our findings support this, showing substantial improvements in hardness with longer exposure times (180–270 s). This is consistent with the principle that polymerization efficiency depends on the total energy delivered (irradiance × exposure time) and aligns with prior studies showing enhanced hardness with prolonged curing (33, 34).

Although extended light-curing times improved microhardness, the potential biological implications of this approach should also be considered. Increasing irradiation duration may increase heat generation within the tooth-restoration complex, potentially elevating intrapulpal temperature. Previous studies have demonstrated that excessive temperature increases may adversely affect pulpal tissues, although the magnitude of this effect depends on factors such as remaining dentin thickness, restoration design, ceramic properties, and the irradiance of the curing unit. Therefore, while prolonged curing may be advantageous for polymerization beneath thick indirect restorations, clinicians should balance these benefits against the potential risk of heat generation and should adhere to manufacturer recommendations whenever possible (19, 20).

While other studies reported no additional benefit beyond 120 s under certain conditions, they attributed this effect to the role of irradiance and the composition of the resin composite material (33, 48), which may explain discrepancies across studies. In our experiment, the combination of higher preheating temperatures, longer preheating durations, and extended photoactivation was associated with higher HV values, potentially through greater crosslinking, resulting in superior HV values.

This effect was most pronounced at the highest preheating temperature and the most extended duration, where enhanced molecular mobility amplified the benefits of prolonged curing. Together, these findings suggest that, among the preheating protocols evaluated, higher temperatures, longer durations, and extended curing times were associated with improved microhardness beneath indirect restorations.

No meaningful correlation was found between HV and DC across most tested groups, which is consistent with previous studies (49, 50). Although HV is often considered an indirect measure of DC, our experimental results did not support this relationship (31, 49). With the exception of one isolated condition, HV and DC were not significantly correlated, suggesting that these properties are largely independent under the tested protocols. This generally weak or non-existent relationship observed may be attributed to the specific properties of the material, as noted in other studies (44). Similar dissociations between hardness and DC under thermal treatment conditions have been reported previously. For example, studies on fiber-reinforced composites demonstrated that preheating significantly increased hardness without producing corresponding changes in DC, suggesting that improvements in mechanical properties may result from increased crosslink density and a more homogeneous polymer network rather than a simple increase in monomer conversion (51).

In addition, the methodology used to assess DC should be considered when interpreting these findings. In the present study, entire specimens were pulverized prior to FTIR analysis, resulting in a bulk average measurement of DC rather than a depth-specific assessment. Several studies have used a similar approach (52–54), and previous investigations have reported no significant differences in DC within the first 2–4 mm^3^ of composite thickness (54). Nevertheless, pulverization may obscure potential surface-to-bulk heterogeneity by averaging the conversion throughout the specimen (49, 50). This is particularly relevant because microhardness was measured on the light-exposed surface of intact specimens, whereas DC reflected the average conversion of the entire specimen. Resin composites are inherently heterogeneous materials in which incident light is scattered at the resin–filler interfaces, and increased filler loading or irregular filler morphology may further reduce light transmission through the material (55). Consequently, the surface, which receives higher irradiance, may undergo more efficient polymerization and exhibit higher hardness values, whereas deeper regions receive attenuated light that may limit monomer conversion (56). Localized increases in surface polymerization or crosslink density resulting from preheating or extended curing may therefore be detected by HV testing but may not be reflected proportionally in the FTIR-derived DC values. This interpretation is supported by a previous study on fiber-reinforced composites, which reported that preheating and post-curing improved hardness without affecting DC, likely due to the formation of a more homogeneous and highly crosslinked polymer network, highlighting that increased hardness does not necessarily correspond to greater monomer conversion (51). Therefore, direct comparisons between HV and DC should be interpreted with caution, and these methodological and material-related factors may partially explain the weak or absent correlation observed between these properties.

Alternative explanations for the present findings should also be considered. The absence of significant differences in DC may indicate that the tested curing protocols already achieved a threshold beyond which additional thermal energy or prolonged photoactivation produced limited gains in monomer conversion. Furthermore, the material-specific composition of the investigated resin composite, including its filler content, photoinitiator system, and resin matrix chemistry, may have influenced its response to preheating, as different composite formulations have been shown to respond differently to preheating protocols (37, 57). Similarly, the increase in microhardness observed with higher preheating temperatures and longer curing times may not be solely attributable to enhanced conversion, but could also reflect changes in polymer network architecture, crosslink density, or post-curing maturation processes. Collectively, these findings suggest that HV and DC may reflect different aspects of material behavior and should not necessarily be considered interchangeable measures when evaluating the effects of preheating and light curing (57). Further research is needed to clarify these relationships.

In our study, HV was measured seven days after resin composite (RC) polymerization, while DC was assessed within one hour post curing. This temporal difference may partially explain the lack of correlation, as HV reflects surface properties that evolve due to post-curing phenomena, such as network relaxation and changes in crosslink density (22, 24). In contrast, DC provides a measure of initial bulk monomer conversion. The current study demonstrates that restorative resin composites (RCs) can be effectively used as luting agents for indirect restorations. Previous studies have reported the favorable bonding performance of preheated restorative composites when used for adhesive cementation of ceramic restorations, supporting their potential clinical applicability as an alternative to conventional luting materials (58). Although previous studies have reported that preheating may reduce viscosity and film thickness (32, 35), the present study was designed to compare different clinically relevant preheating protocols rather than preheated and non-preheated resin composites. Within the investigated conditions, higher preheating temperatures, longer preheating durations, and extended light-curing times were associated with significantly higher microhardness (HV) beneath thick ceramic restorations. This mechanical improvement, however, depends on a streamlined workflow with minimal delay between composite removal from the heating device and light activation. The findings highlight the importance of optimizing preheating and curing protocols when restorative RCs are used as luting agents beneath thick ceramic restorations. Although the degree of conversion (DC) remained unaffected across the investigated conditions, the improved microhardness observed under specific preheating and curing protocols supports the potential use of restorative RCs as luting agents, provided that appropriate handling and polymerization procedures are followed.

From a clinical perspective, the present findings suggest that restorative resin composites used under the investigated preheating conditions may represent a viable alternative to conventional resin cements when luting thick indirect ceramic restorations. The improved microhardness observed with higher preheating temperatures, longer preheating durations, and extended light-curing times may contribute to enhancing the mechanical performance and durability of the luting material. However, the practical implementation of these protocols requires careful consideration. Achieving and maintaining elevated composite temperatures during restoration placement may be challenging in routine clinical practice, particularly when multiple procedural steps are required before light activation. Furthermore, the extended curing times identified as beneficial in this study (180–270 s) should be interpreted as optimal experimental conditions for the investigated materials and restoration configuration rather than universally recommended clinical protocols. The clinical applicability of such curing durations may vary depending on restoration thickness, ceramic properties, curing unit characteristics, and patient-specific considerations. In addition, these prolonged curing times may increase chairside treatment time and may not always be feasible in busy clinical settings. Therefore, clinicians must balance the potential mechanical benefits observed under these experimental conditions against the additional time, equipment, and workflow requirements needed to achieve them. Future clinical studies are required to determine whether the improvements observed *in vitro* translate into meaningful long-term benefits for restoration survival and clinical performance.

The present findings may have practical implications for clinicians using preheated resin composites as luting agents beneath thick ceramic restorations. Based on the experimental conditions investigated, several evidence-based recommendations can be proposed to optimize polymerization outcomes and microhardness under clinically relevant conditions. These recommendations are summarized in Table 5.

**Table 5:** Clinical recommendations based on the present findings.

Clinical parameter	Recommendation	Clinical rationale
Preheating temperature	68 °C preferred over 54 °C among the conditions evaluated	Higher microhardness values were observed at 68 °C.
Preheating duration	15 min preferred over 5–10 min	Longer preheating duration resulted in significantly greater microhardness.
Light-curing duration	90 s from each of the buccal, occlusal, and lingual directions (total curing time: 270 s)	Extended multidirectional curing was associated with the highest microhardness values beneath 4 mm lithium disilicate restorations.
Delay before curing	Minimize delay between removal from the heater and light activation	Temperature loss may reduce the benefits of preheating and potentially diminish the positive effects on polymerization.

Recommendations are limited to the materials, preheating temperatures (54 °C and 68 °C), preheating durations (5–15 min), and curing protocols investigated in the present study.

This was an *in vitro* study simulating clinical conditions wherein the luting agent was light-cured beneath a thick anatomic ceramic restoration. However, several limitations should be considered. Only one resin composite (of a single brand and shade) and one ceramic material (low-translucency lithium disilicate, 4 mm thickness) were tested (15). Since both the resin composition/shade and ceramic type/thickness can significantly influence light transmission and polymerization (39), the findings may not be generalizable to other materials or clinical scenarios.

Another limitation is that the study was conducted in a single experimental setting by a single operator. Although all procedures were performed according to a standardized protocol to minimize variability, operator-dependent bias cannot be completely excluded. In addition, the controlled laboratory environment may not fully replicate the variability encountered in clinical practice, where differences in operator technique, workflow, and patient-related factors may influence the performance of preheated resin composites. Therefore, caution should be exercised when extrapolating these findings directly to clinical settings.

To replicate clinical handling, a relatively long delay (up to 60 s) was allowed between RC preheating and light curing, reflecting the time required for restoration placement and excess removal. During this interval, the composite inevitably cooled down in a time-dependent manner (59, 60), which may have diminished the benefits of preheating; however, real-time temperature changes were not measured, and the assumption of cooling was based on prior studies (42, 61). The Spearman correlation analyses were conducted within subgroups of *n* = 5 per cell, yielding wide confidence intervals and limited statistical precision. All correlation findings should therefore be interpreted as exploratory rather than confirmatory, and the single significant result should be interpreted with caution.

An additional limitation relates to sample size and statistical power. Although the sample size exceeded the minimum requirement calculated from the effect size derived from the reference study, the use of five specimens per group may limit the ability to detect small-to-medium effects under more conservative assumptions. This is particularly relevant for the degree of conversion outcome, for which no significant effects were observed. Therefore, while the findings suggest that the investigated variables did not significantly influence degree of conversion, the possibility of small effects cannot be completely excluded. Future studies with larger sample sizes may help further clarify these relationships.

The present study was designed to compare clinically relevant preheating protocols rather than to evaluate preheated vs. non-preheated resin composites. Consequently, a room-temperature control group was not included. Therefore, the findings should be interpreted as comparisons among different preheating temperatures, durations, and light-curing protocols, and no conclusions can be drawn regarding the effect of preheating itself relative to non-preheated composites. Future studies incorporating a room-temperature control group would be valuable in order to quantify the overall bene

Future research should further investigate both the mechanistic and clinical aspects of preheated resin composites. Incorporating real-time temperature monitoring would provide quantitative data on temperature loss during clinical handling and help determine the optimal interval between preheating and light activation. The development and evaluation of thermally controlled composite delivery systems, such as self-heating syringes, may help maintain elevated temperatures during placement and reduce heat loss prior to polymerization.

Future studies should also evaluate the influence of material-related factors, including composite shade, translucency, filler content, and photoinitiator systems, on the response to preheating (62). In addition, comparisons among microhybrid, nanohybrid, and bulk-fill resin composites under standardized preheating and curing protocols are needed to determine whether the observed effects are material-specific.

Finally, long-term aging studies and randomized clinical investigations are required to establish whether the improvements in microhardness observed *in vitro* translate into meaningful clinical benefits, including enhanced restoration longevity and performance.

## 5 Conclusions

Within the limitations of this *in vitro* study, resin composites preheated at 68 °C for 15 min exhibited significantly higher microhardness than those preheated at lower temperatures or for shorter durations, particularly when combined with extended light-curing times (180–270 s) beneath 4 mm lithium disilicate restorations. In contrast, degree of conversion remained unaffected by the investigated preheating temperatures, preheating durations, and light-curing protocols.

Accordingly, the first null hypothesis was not rejected, as preheating temperature, preheating duration, and light-curing time did not significantly affect the degree of conversion. The second null hypothesis was rejected because these factors significantly influenced microhardness. The third null hypothesis was not rejected, as no significant association was observed between degree of conversion and microhardness.

These findings indicate that, among the preheating protocols evaluated, higher preheating temperatures, longer preheating durations, and extended light-curing times were associated with increased microhardness. Because a non-preheated control group was not included, the results should be interpreted as comparisons among different preheating and curing protocols rather than evidence of the benefits of preheating itself. Within the investigated conditions, optimization of preheating and curing protocols may improve the mechanical performance of resin composites used as luting agents beneath thick ceramic restorations.

## Data Availability

The raw data supporting the conclusions of this article will be made available by the authors, without undue reservation.
